# Trouble on the Farm

**Published:** 1902-01

**Authors:** Holman F. Day


### TROUBLE ON THE FARM.

BY HOLMAN F. DAY.

(Copyright, Walter B. Guild, 1901.)

Just as soon as things are splendid an' I'm gittin' some ahead,
An' the crops are lookin' scrumptious an' the stock is all well-fed,
When I've got it nicely figured what the profits ought to be,
Then all to once disaster comes a-swoopin' down on me.
Fightin' 'tater-bugs an' squash-bugs, beetles, worms an' like o' that
Has swamped me pretty often an' has mowed the profits flat,
But I've never done much kickin', for we farmers, don't ye know,
Have allus made allowance for a sartin dose of woe.
For it's fair an' open warfare on the beetles, bugs an' sich,
Ye can give the critters tophet with your pizen, punk an' pitch;
Ye can smash 'em when ye find 'em; but the things that wallops me
Are the pesky new contraptions that ye never, never see.

I owned a pair of hosses, sixteen hands an' strong's a beam—
There wa'n't in all our section sich a hefty, spiffy team—
They were mighty good in haulin', they were spry upon the road,
An' there wa'n't an out about 'em, insofar as what I knowed.
But all to once, by thunder, came a vet'rinary cuss,
An' he looked them hosses over an' he up an' made a fuss.
He had a little spy-glass an' he squinted with one eye,
An' he said they had the glarnders, 'cause he found tobackilli.
So he promptly up an' shot 'em, an' I never got no pay;—
I'm a pretty patient critter, as my neighbors all will say,
But that action made me ugly, for the things that worry me
Are these pesky new contraptions that ye never, never see.

I owned some harnsome Sussex shotes, so fat they couldn't squeal,
I fed 'em corn an' middlin's an' I topped 'em off on meal;
They was sure three-hundred pounders—would have brought a fancy price—
An' I bragged a lot about 'em; they was sartin extry nice.
Wal, at last I had 'em butchered, an' I took 'em down to sell,
But, by Judas, them inspectors came around to peek an' smell,
An' they said I mustn't sell 'em—I must bury 'em, because
They were full o' tritchernoses! 'S if I knowed what them things was!
I can fight these bugs an' beetles, but may tophet swaller me
If I'm good for new contraptions that ye never, never see.

Wal, I had a herd of Jerseys—sleek an' fat an' all o' that—
Owned a milk route to the city an' was clearin' quite a slat;
Milk was thick an' nice an' yaller, an' I sold it ev'ry drop.
One day, though, a blamed inspector climbed aboard an' made me stop.
Took his dinky little spy-glass an' he peeked an' peeked a while,
An' he curled his lip an' snorted an' says he, "That milk is vile!"
Then says I, as mad as thunder, "What's the matter, if ye please?"
"There is tookerboobles in it, an'," says he, "they're thicker'n fleas!"
Do ye wonder, friends an' neighbors, that my Ichabod was stirred?
But it didn't make no diff'rence, for they come an' killed my herd.
Blame the luck! Gol swat their notions! Won't they ever let me be—
Findin' out these new contraptions that ye never, never see?

